# Notices, Varieties, &c.

**Published:** 1858-09

**Authors:** 


### NOTICES, VARIETIES, &c.

*The British and Foreign Medico-Chirurgical Review*, No. 43, for July, 1858, reprinted by the Messrs. WOOD, 389 Broadway, New York, is, we think, one of the most valuable numbers recently published ; it contains forty-six different articles—(that of SALTER on Asthma, 16 pp. 8vo., should be purchased, and read, and studied, by every physician, old or young, in all lands). $3 a year. It contains a list of eighty-six new medical publications from Europe, imported by the Messrs. W., besides thirty-six "recent" American works.

*North-Western Farmer*, monthly, one dollar a year, Dubuque, Iowa, is well worthy of the patronage of Agriculturists in the great North-West.

*The Mothers' Journal*, New York, edited by Mrs. CAROLINE O. HISCOX—monthly, one dollar a year—we commend to all families loving solid, practical reading.

*Ladies' Home Magazine*, three dollars a year, Philadelphia, is, like all its predecessors, full of safe and profitable reading for our wives and daughters.

*Sargent's School Monthly*, Boston, one dollar a year, is a valuable family periodical. Old and young can read it to advantage. We wish every family could take it, and practice its precepts on reading, conversation, and kindred subjects.

*Hall's Journal of Health*, New York, one dollar a year, is oftener quoted than any monthly publication in the world. It is more praised, and less blamed, than any periodical we know of; it is one of that very small class of papers whose subscription list was doubled in the late commercial revulsion, and is daily growing larger, without pufferies, agencies, or advertisings. It has not a delinquent subscriber. We believe it has not a single reader who does not wish for its increased distribution; and, not least, it is a "paying concern:" the worse the times, the greater the profits. It is doing a moral and medical good wherever it goes; and we hope that its Editor will live many years, to teach and secure the practice of what is true, useful, and good !

*The Happy Home,* monthly, one dollar a year, Boston—enriched with engravings, paintings, and music—begins its August number with "Family Scenes of the Bible." No. 8. Extravagance in Dress, Editorial: A Lesson for Young Men, by Mrs. C. W. ALLEN; Washing-day; Secret of being Happy, &c.—all good, and of practical interest.

*Remarks on the Medicinal Hypophosphates*, by WILLIAM PROCTOR, Jr., is a pertinent article in the July number of the *Eclectic Medical Journal* of Philadelphia, monthly, two dollars a year.

*Merry's Museum*, one dollar a year, New York, has entered its thirty-sixth volume—proof positive of its appreciation by multitudes of young folks all over Uncle Sam's dominions. The August number abounds with interesting, and safe, and valuable reading.

*Presbyterian Expositor*, Chicago, one dollar and fifty cents a year—edited by that distinguished polemic and able divine, Rev. L. N. RICE, D. D.—is winning its way to its deserved and high estimation among the friends of Presbytery.

*The Scientific Artisan*, Cincinnati, No. 1, promises, in mechanical beauty, and in substance matter, to rival our own *Scientific American* in New York, which has so long been a favorite among inventors and educated men. Whether it will keep the promise of its beginning, time alone will tell.

*Christian Review*, Baltimore—a Baptist quarterly, at three dollars a year—has several articles for July, largely worth the study of educated and elevated minds. Among these are: The Religion of Phrenology; Alleged Discrepancies in the Bible; Christianity in the Legal Profession.

*Consumption*, by E. H. STOCKWELL, M. D., reads very much like a publication entitled "*Consumption*, by W. W. HALL, New York." Either HALL was read by STOCKWELL, or STOCKWELL read HALL, we can't say which; at all events, their sentiments correspond in many essential points; and we are glad that Dr. S. is engaged in the dissemination of true views on this most important subject.

"*Economy of Food; or, What shall we Eat?* Being Useful Lessons for Rich and Poor, including the Story of 'A Dime a Day;' Showing How 'twas Earned, and How 'twas Spent, and How Five Mouths it Fed"—is a publication which all may read with profit. It shows how the rich might live more comfortably and happy on a great deal less, and consequently have a great deal more to give to suffering humanity; and, not less important, it teaches the poor, by example and fact, the lesson, ignorance of which keeps so many of them poor to the last day of life, how to economize, how to save, and how to lay up for a rainy day, even out of a scanty income. The want of food, and ignorance of the proper method of preparation, is the source of an incalculable amount of disease. And with a view to promote the circulation of this useful publication of SOLON ROBINSON, at our own cost, we will send a copy, without charge, to every new subscriber who desires it, during September and October.

*Blackwood's Edinburgh Magazine*, monthly—republished, at three dollars a year, by LEONARD SCOTT & Co., New York—is now in its forty-sixth volume. Its contributors are among the best writers in Great Britain—treating on subjects of historical, scientific, political, and general interest.

The successful laying of the Atlantic cable brings immortal honor to MORSE and FIELD, and our own Kentucky HUGHES—little Jersey, too, must come in for her share, through her wise, humane, and noble-hearted PETER COOPER; hence, we give a loud hurrah for New York, Massachusetts, Jersey, and old Kentuck—a quarternity in one, all essential, each indispensable: for while MORSE invented the cable, and PETER COOPER's money largely helped to make it, the unflinching energy and undaunted courage of CYRUS W. FIELD was essential to its deposition at the bottom of the sea; but to make it easily and largely available after all this was done, the invention of Mr. HUGHES fits bravely in, and a world's triumph is complete. Shouldn't be greatly surprised, if some sunny morning, among the letters or telegrams laid on our table, one should come by the cable, with a summons to attend the bedside, to remove the cough of Lord SMITH, EARL BROWN, or the Duchess of Tweedledee. That a medical summons should come across the water, for such men as Surgeon CARNOCHAN, HORACE GREEN, or VALENTINE MOTT, men of a just and world-wide renown, is a very probable event.

*Errors of Education*, an Address by Rev. J. C. BAYLESS, of Kentucky, and published by request, is one among other signs of the times, that men of education, of culture, and of influence, are waking up the masses to the importance of physical education, combined with the literary and the moral.

OFFER.—We will give any two numbers of HALL'S JOURNAL OF HEALTH, for each number for January, 1848, sent post-paid to Dr. W. W. HALL, New York, during the month of September; after that, we will have a new supply of all our back numbers.

*Country Gentleman*, Albany, New York, two dollars a year, if paid in advance, is the most beautiful, comely, and valuable weekly agricultural in the United States.

*Godey's Lady's Book and Graham's Magazines*, for September, are already received, and will meet with a welcome from their multitudinous patrons. Three dollars a year, each; Philadelphia.

*American Railway Guide*, 9 Spruce street, New York, corrected every month, gives the fare, distance, time of starting, &c., on all established routes by river, railway, or steamship, in the United States—of great value to business men and travellers; twenty-five cents each.

THE only medical publication in the world which never advises a dose of physic, nor expensive contrivances or appliances, HALL'S *Journal of Health*, New York, monthly. One dolla[illegible] year. All subscriptions begin with January.

